# Comparison of human corneal cell density by age and corneal location: an in vivo confocal microscopy study

**DOI:** 10.1186/s12886-016-0290-5

**Published:** 2016-07-16

**Authors:** Tianyu Zheng, Qihua Le, Jiaxu Hong, Jianjiang Xu

**Affiliations:** Department of Ophthalmology, EYE and ENT Hospital of Fudan University, 83 Fenyang Road, Shanghai, 200031 China

**Keywords:** Morphology, Confocal microscopy, Cornea, Central, Peripheral, Age

## Abstract

**Background:**

Peripheral and central regions of the cornea are optically different and have different repair capacity and pathology. For this reason, we characterized the cellular morphology and quantified the cell density of the central and peripheral regions of the cornea with age.

**Methods:**

Eighty healthy subjects were enrolled in the study and divided into four groups according to age: A (0–19 years), B (20–39 years), C (40–59 years), and D (>60 years). In vivo confocal microscopy was used to measure the following parameters for the central and peripheral regions of the cornea: average cellular density and area of the superficial and basal epithelium; average density of the anterior and posterior keratocytes; average endothelial cell density and cellular area; percentage of hexagonal endothelial cells.

**Results:**

Statistically significant differences between the central and peripheral cornea were observed for the cellular density of basal epithelial cells in group A. The density of keratocytes in the anterior stroma was significantly greater in the central region compared with the peripheral region in group B and group C. The percentage of hexagonal cells in the endothelial layer was significantly greater in the central region compared with the peripheral region. Age-related changes were found in peripheral basal epithelial cell density, central and peripheral endothelial cell density, and the percentage of hexagonal endothelial cells.

**Conclusion:**

Both similarities and differences in morphology of the central and peripheral regions of the transparent cornea were observed. These observations would provide a histological basis for further studies to define its regional pathological mechanisms.

## Background

Although no clear boundary could be identified between the central and peripheral transparent cornea, these two regions are anatomically and physiologically different in thickness, keratometry, nutritional sources, biochemical composition and disease susceptibility [1–3]. The central cornea is optically superior to the peripheral region [4, 5], whereas the peripheral cornea has a greater repair capacity [6, 7]. Moreover, corneal edema occurs more frequently in the central region than in the peripheral region [8], infectious diseases are commonly seen in the central region of the cornea, and the peripheral region is usually affected by immunological disease [9].

Confocal microscopy allows an in vivo examination of the human cornea at the cellular level. In vivo corneal confocal microscopy provides noninvasive, real-time images with high resolution and contrast of all layers in both normal and diseased corneas [10]. However, at present, no histological or in vivo morphological study has addressed the cellular differences between the central and peripheral regions of the cornea in humans, which might be a basis for the optical, physiological and pathological differences between these two regions. Previous studies have focused on the central cornea and demonstrated age-related cellular changes in the density of keratocytes [11, 12] and the density and size of endothelial cells [11, 13, 14]. In the present study, we used in vivo confocal microscopy (CM) to explore the morphology of the central and peripheral cornea, measure the cellular density of each layer, and evaluate the similarities and differences between these two regions with age.

## Methods

### Study subjects

Eighty healthy volunteers with a mean age of 39 ± 23 years were recruited in the EYE and ENT Hospital of Fudan University, Shanghai between March and July of 2015. Inclusion criteria were: no history of ocular surgery, trauma, infection or use of contact lenses; slit lamp and ophthalmoscope examinations were both normal. By slit lamp examination, we measured corneal fluorescence staining, tear film break up time (BUT) and tear meniscus height (TMH) to exclude the dry eye. Subjects with pterygium or pinguecula, corneal fluorescence staining, BUT < 10s or a TMH < 0.2 mm were excluded, according to the TMH measurements of normal eyes (0.19 ± 0.09 mm) [15]. Subjects were grouped by age (Table 1).

**Table 1:** Group demographics

Parameter	Group A	Group B	Group C	Group D
Average Age (*y*)	13 ± 4	29 ± 6	49 ± 6	74 ± 7
Age Range (*y*)	0–19	20–39	40–59	60–79
Males (*n*)	12	11	12	8
Females (*n*)	8	9	8	12

### In vivo confocal microscopy

Examination by in vivo CM (Confoscan 3.0, Nidek, Japan) was performed on the right or left eye chosen using a random number table. After administering one drop of topical anesthesia (Benoxil, 0.4% oxybuprocaine; Santen Pharmaceutical Co., Japan), the patient was asked to place their chin and forehead firmly on the headrest of the microscope. A drop of Vidisic gel (0.2% Carbomer 940; Bausch and Lomb, Germany) on the non-applanating lens (Achroplan, 409/0/75W; Zeiss, Germany) served as an immersion and contact substance. To obtain confocal images of the cornea, the front surface of the immersion lens was manually advanced with a joystick hand piece attached to the main body of the instrument until sharp images of the cornea were visualized on the monitor. There was no direct contact between the corneal surface and objective lens, which had a concave surface and a working distance of 1.9 mm. Serial optical sections covered a field of approximately 300 × 400 μm and a z-axis optical slice of 3.2 μm. To acquire images of the central cornea, the subject was required to look straight. Then the subjects were asked to look upward to examine the inferior peripheral corneal region which is 2–3 mm inside the inferior limbus. During a single examination, approximately 350 sequential digital images were obtained from the endothelium to the superficial epithelium, and were directly saved into the hard disk drive.

### Statistical analysis

Five images of each layer were randomly chosen, and the built-in software (Navis; Lucent Technologies, Murray Hill, NJ) was used to measure the average cellular density and area of the superficial and basal epithelium, the average density of anterior and posterior stroma cells, and the average endothelial cell density, cellular area and percentage of hexagonal cells in the central and peripheral cornea. In accordance with a previous study [11], the anterior stroma was defined as the first three clear images (without motion blur or compression lines) immediately posterior to Bowman’s layer, within 50 μm posterior to Bowman’s layer. The posterior stroma was defined as the first three clear images immediately anterior to Descemet’s membrane, within 50 μm anterior to Descemet’s membrane. There was no missing data on any variable of any subject. Differences between the central and peripheral cornea were tested using the paired Student’s *t*-test. Age-related changes among different age groups were analyzed by one-way analysis of variance (ANOVA) and the least significant difference (LSD) test. *P* < 0.05 was considered statistically significant.

## Results

### Epithelial cells

**Fig. 1:**
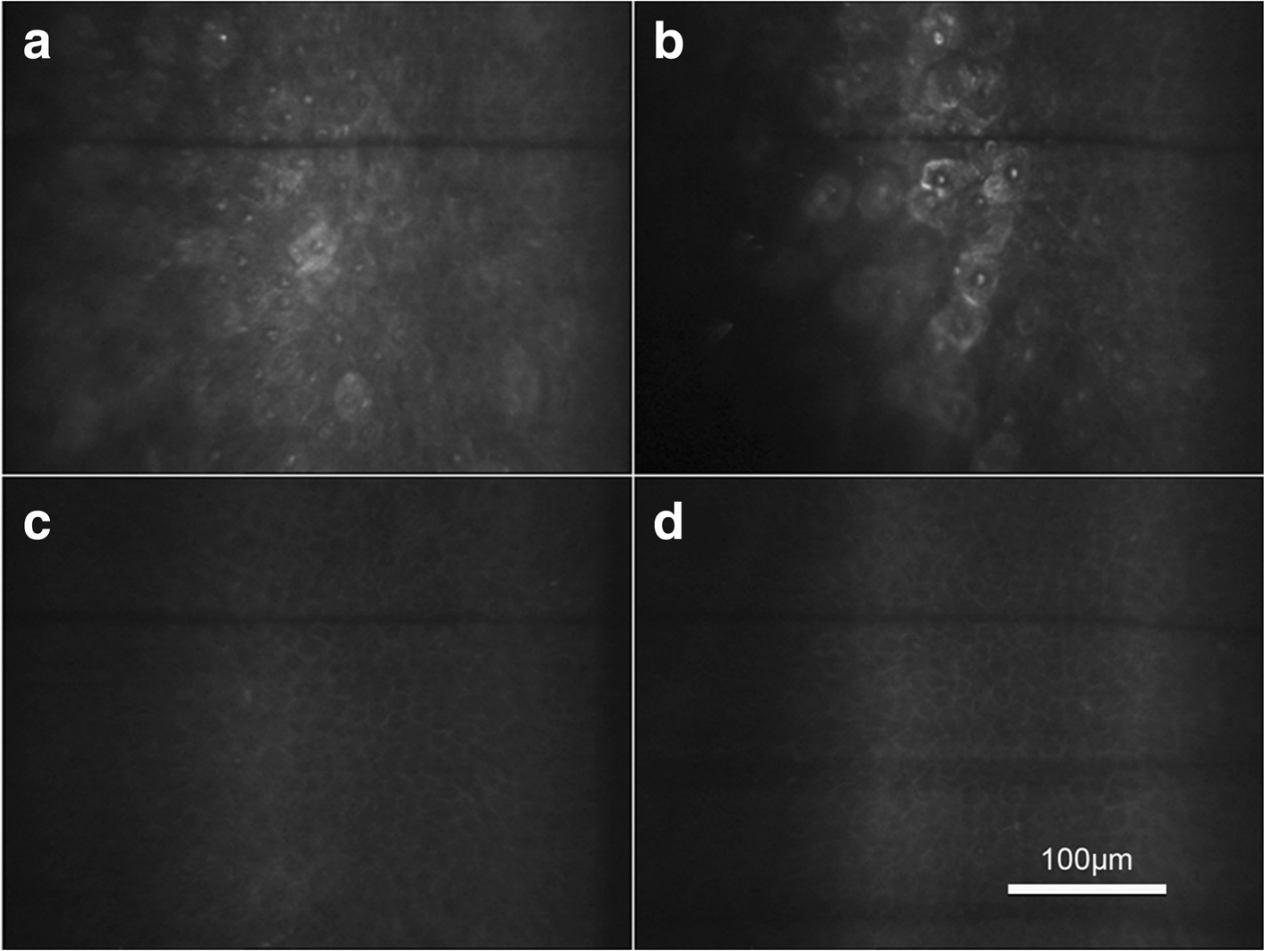
“In vivo confocal micrographs of the clear cornea showing the epithelium at the central and peripheral transparent cornea”. Caption: **a** Superficial epithelial cells in the central cornea were large and polygonal with bright cell boundaries and discernable nuclei. **b** Superficial epithelial cells in the peripheral cornea with the same morphology as (**a**). **c** Basal epithelial cells in the central cornea in a regular dense arrangement with smaller cell bodies. **d** Basal epithelial cells in the peripheral cornea with the same morphology as (**c**)

**Fig. 2:**
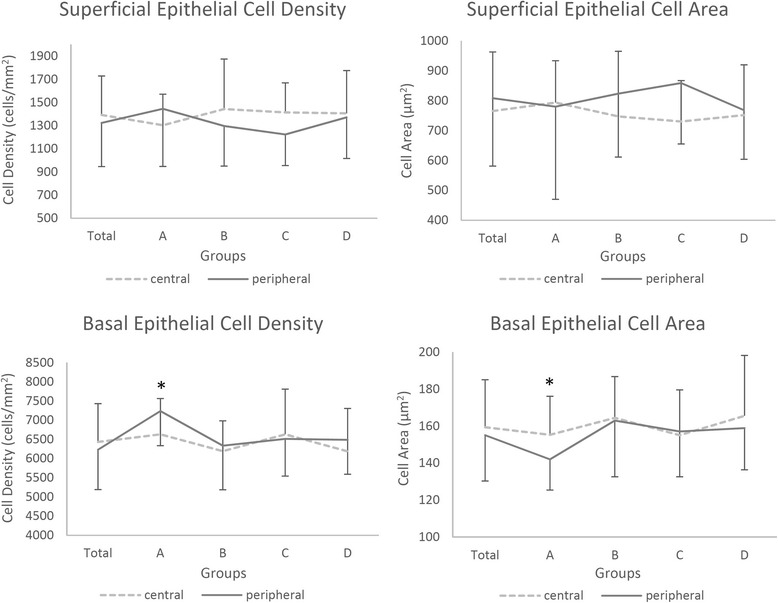
“Superficial and basal epithelial cell density and area in the central and peripheral transparent cornea”. Caption: Group A: 0–19 years old; Group B: 20–39 years old; Group C: 40–59 years old; Group D: 60–79 years old. *Significant difference between the central and peripheral regions (*P* < 0.05; ---- central region, ▬ peripheral region). Bars represent the standard deviation (up-direction: central region; down-direction: peripheral region)

The morphology of epithelial cells between the central and peripheral cornea was not significantly different. Superficial epithelial cells were large and polygonal, with bright cell boundaries and discernable nuclei (Fig. 1). There were no age-related differences in superficial epithelial cell density (central: *F* = 0.553, *P* = 0.648; peripheral: *F* = 1.515, *P* = 0.219) and area (central: *F* = 0.186, *P* = 0.905; peripheral: *F* = 0.552, *P* = 0.649) (Fig. 2). Neither superficial epithelial cell density nor cell area were significantly different between the central and peripheral regions (*P* > 0.05, Table 2 and Fig. 2).

**Table 2:** Superficial epithelial cell density and area in the central and peripheral transparent cornea

	Density (cells/mm^2^)	Area (μm^2^)
Total		
Central	1392 ± 337	766 ± 197
Peripheral	1323 ± 377	808 ± 227
*t* value	1.191	1.165
*P* value	0.238	0.248
Group A		
Central	1303 ± 268	794 ± 140
Peripheral	1445 ± 498	780 ± 310
*t* value	1.278	0.174
*P* value	0.219	0.864
Group B		
Central	1443 ± 432	748 ± 218
Peripheral	1296 ± 346	824 ± 212
*t* value	1.164	1.112
*P* value	0.261	0.282
Group C		
Central	1414 ± 255	730 ± 137
Peripheral	1223 ± 268	859 ± 204
*t* value	2.022	1.002
*P* value	0.06	0.06
Group D		
Central	1406 ± 369	752 ± 168
Peripheral	1371 ± 355	768 ± 164
*t* value	0.27	0.289
*P* value	0.791	0.777

Differences between the central and peripheral cornea were analyzed using the paired Student’s *t*-test (*P* < 0.05 was considered statistically significant)

Basal epithelial cells were in a regular dense arrangement with smaller cell bodies compared with superficial cells. The nuclei could hardly be discriminated despite clear cellular boundaries (Fig. 1). Age-related changes in epithelial cell density of the peripheral region were significant (*F* = 3.196, *P* = 0.028), but changes in the basal epithelium of the central region were not significant (*F* = 1.250, *P* = 0.298). In group A, basal epithelial cell density of the peripheral region was significantly higher compared with the other three groups, while there were no significant differences among these three groups (Fig. 2). In group A, basal cell density of the peripheral region was higher compared with the central region (*t* = 2.225, *P* = 0.038; Table 3 and Fig. 2). There were no significant differences between basal cell densities of the central and peripheral regions in other age groups or in the total subjects (*P* > 0.05, Table 3 and Fig. 2).

**Table 3:** Basal epithelial cell density and area in the central and peripheral transparent cornea

	Density (cells/mm^2^)	Area (μm^2^)
Total		
Central	6433 ± 999	159 ± 26
Peripheral	6628 ± 1038	155 ± 25
*t* value	1.359	1.177
*P* value	0.178	0.243
Group A		
Central	6632 ± 932	155 ± 21
Peripheral	7238 ± 903	142 ± 17
*t* value	2.225	2.323
*P* value	0.038*	0.032*
Group B		
Central	6193 ± 789	164 ± 22
Peripheral	6334 ± 1148	163 ± 30
*t* value	0.521	0.204
*P* value	0.608	0.84
Group C		
Central	6,633 ± 1177	155 ± 24
Peripheral	6513 ± 972	157 ± 24
*t* value	0.395	0.26
*P* value	0.697	0.798
Group D		
Central	6189 ± 1115	166 ± 33
Peripheral	6488 ± 898	159 ± 22
*t* value	0.978	0.773
*P* value	0.344	0.451

Differences between the central and peripheral cornea were analyzed using the paired Student’s *t*-test (*P < 0.05 was considered statistically significant)

### Keratocytes

**Fig. 3:**
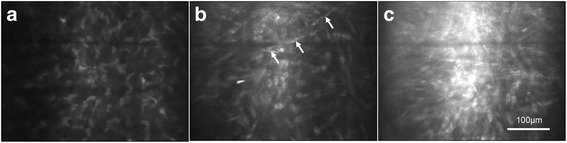
“In vivo confocal micrographs of the clear cornea showing the corneal stroma at the central and peripheral regions”. Caption: **a** Keratocytes in the central cornea were hyper-reflective spindle- or osteoblast-shaped with a uniformly dark background. **b** Keratocytes in the peripheral cornea with a cellular morphology similar to those in the central part, but some needle-shaped hyper-reflective deposits (arrow) could be identified in the background. **c** Keratocytes could hardly be discriminated and the reflection of stromal fibers was increasingly bright from the corneal stroma to scleral fibers

Keratocytes in both the central and peripheral regions of the cornea were hyper-reflective spindle- or osteoblast-shaped cells. However, they stood out against the uniform dark background in the central cornea, while some needle-shaped hyper-reflective deposits could be seen in the background of the peripheral cornea. In transitioning from the stroma to the limbus, the reflection of stromal fibers became increasingly brighter (Fig. 3).

**Fig. 4:**
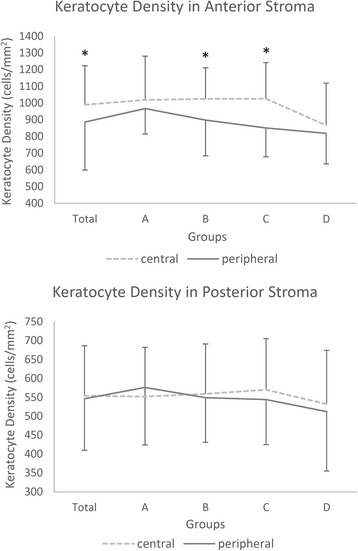
“Anterior and posterior keratocyte density in the central and peripheral transparent cornea”. Caption: Group A: 0–19 years old; Group B: 20–39 years old; Group C: 40–59 years old; Group D: 60–79 years old. *Significant difference between the central and peripheral regions (*P* < 0.05; ---- central region, ▬ peripheral region). Bars represent the standard deviation (up-direction: central region; down-direction: peripheral region)

No age-related changes in keratocyte density were observed between the anterior or posterior stroma from the central or peripheral regions of the cornea (anterior-central: *F* = 1.973, *P* = 0.126; anterior-peripheral: *F* = 2.247, *P* = 0.090; posterior-central: *F* = 0.256, *P* = 0.857; posterior-peripheral: *F* = 0.652, *P* = 0.584; Fig. 4). The difference between keratocyte density in the anterior stroma of the central and peripheral regions was statistically significant for all subjects (*t* = 3.37, *P* = 0.001; Table 4 and Fig. 4). Further analysis revealed that keratocyte density in the anterior central cornea was significantly higher compared with the peripheral region in groups B and C (Group B: *t* = 2.161, *P* = 0.043; Group C: *t* = 3.612, *P* = 0.002; Table 4 and Fig. 4). Keratocyte density of the posterior stroma was not significantly different between the central and peripheral regions of the cornea in all subjects or in each group (Table 4 and Fig. 4).

**Table 4:** Keratocyte density in the central and peripheral transparent cornea

Parameters	Anterior stroma (cells/mm^2^)	Posterior stroma (cells/mm^2^)
Total		
Central	990 ± 233	554 ± 132
Peripheral	886 ± 187	546 ± 136
*t* value	3.37	0.435
*P* value	0.001*	0.665
Group A		
Central	1,019 ± 262	552 ± 130
Peripheral	967 ± 152	576 ± 152
*t* value	0.813	0.764
*P* value	0.427	0.455
Group B		
Central	1,025 ± 186	559 ± 132
Peripheral	898 ± 214	549 ± 118
*t* value	2.161	0.333
*P* value	0.043*	0.743
Group C		
Central	1,026 ± 216	570 ± 135
Peripheral	851 ± 173	544 ± 119
*t* value	3.612	0.632
*P* value	0.002*	0.535
Group D		
Central	867 ± 252	532 ± 142
Peripheral	819 ± 183	512 ± 157
*t* value	0.604	0.491
*P* value	0.555	0.630

Differences between the central and peripheral cornea were analyzed using the paired Student’s *t*-test (*P < 0.05 was considered as statistically significant)

### Corneal endothelium

**Fig. 5:**
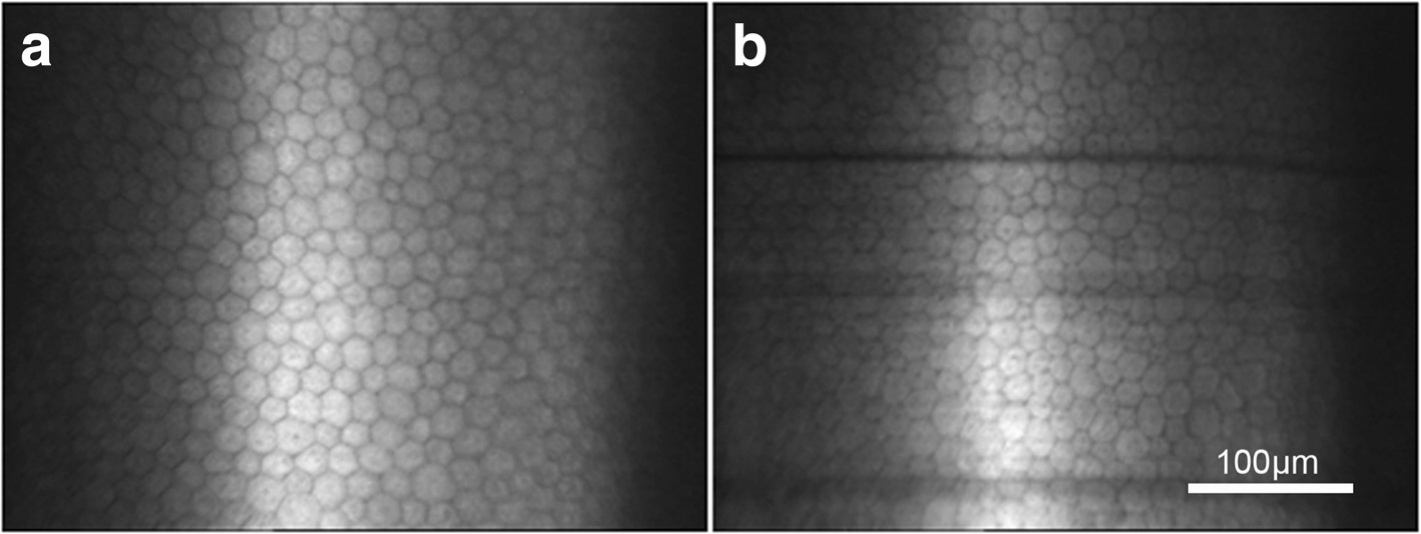
“In vivo confocal micrographs of the clear cornea showing the endothelium at the central and peripheral regions”. Caption: **a** Endothelial cells in the central cornea had a uniform cell size with a regular hexagonal arrangement. **b** Endothelial cells in the peripheral cornea had greater heteromorphism compared with the central cornea

**Fig. 6:**
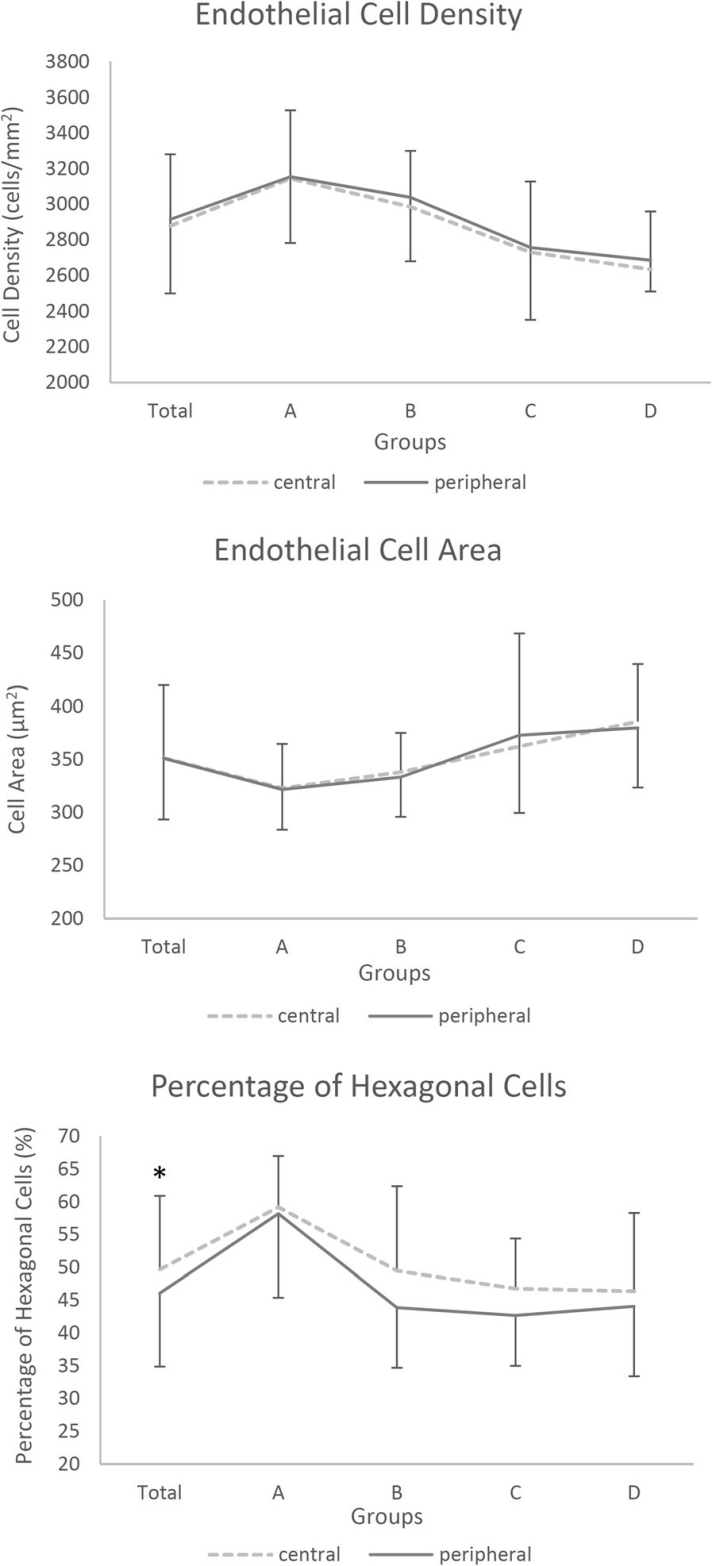
“Endothelial cell density and area, and the percentage of hexagonal cells in the central and peripheral regions of the transparent cornea”. Caption: Group A: 0–19 years old; Group B: 20–39 years old; Group C: 40–59 years old; Group D: 60–79 years old. *Significant difference between the central and peripheral regions (*P* < 0.05; ---- central region, ▬ peripheral region). Bars represent the standard deviation (up-direction: central region; down-direction: peripheral region)

Although endothelial cells were hexagonal in both the central and peripheral regions of the cornea, cell heteromorphism in the peripheral region was greater, compared with the central region of the cornea (Fig. 5). Endothelial cell density and the percentage of hexagonal cells significantly decreased with age in both the central and peripheral regions (cell density-central: *F* = 8.437, *P* < 0.001; cell density-peripheral: *F* = 2.982, *P* = 0.037; hexagonal percentage-central: *F* = 4.782, *P* = 0.005; hexagonal percentage-peripheral: *F* = 8.661, *P* < 0.001; Fig. 6). Average endothelial cell density and area in the central and peripheral cornea were not significantly different (*P* > 0.05, Table 5 and Fig. 6). However, the percentage of hexagonal cells in the central cornea was significantly higher compared to the peripheral region (*t* = 2.189, *P* = 0.032; Table 5 and Fig. 6).

**Table 5:** Endothelial cell density, cell area and the percentage of hexagonal cells in the central and peripheral transparent cornea

Parameters	Density (cells/mm^2^)	Area (μm^2^)	Percentage of hexagonal cells (%)
Total			
Central	2,879 ± 402	351 ± 68	50 ± 11
Peripheral	2,915 ± 417	351 ± 58	46 ± 11
*t* value	1.07	0.059	2.189
*P* value	0.288	0.953	0.032*
Group A			
Central	3,145 ± 383	323 ± 42	59 ± 8
Peripheral	3,155 ± 373	322 ± 38	58 ± 13
*t* value	0.175	0.211	0.211
*P* value	0.863	0.835	0.837
Group B			
Central	2,986 ± 314	338 ± 37	49 ± 13
Peripheral	3,039 ± 360	333 ± 38	44 ± 9
*t* value	1.002	0.824	1.474
*P* value	0.329	0.42	0.16
Group C			
Central	2,730 ± 398	362 ± 106	47 ± 8
Peripheral	2,756 ± 404	373 ± 73	43 ± 8
*t* value	0.398	0.576	1.599
*P* value	0.695	0.572	0.127
Group D			
Central	2,634 ± 326	386 ± 54	46 ± 12
Peripheral	2,685 ± 176	380 ± 56	44 ± 11
*t* value	0.541	0.398	0.972
*P* value	0.596	0.696	0.348

Differences between the central and peripheral cornea were analyzed using the paired Student’s *t*-test (*P < 0.05 was considered statistically significant)

## Discussion

As stated in the Introduction, there are many differences in the anatomy, physiology and pathogenesis of cells in the central and peripheral regions of the transparent cornea [1–9]. The present study confirms that the central and peripheral regions of the cornea had a similar microstructural arrangement, while some differences exist in cell morphology and density in each layer.

### Corneal epithelial cells

Cell density of the superficial corneal epithelium was 1,213 ± 370 cells/mm^2^ in the central region, which is in agreement with a previous study [16]. Cell morphology and density were not significantly different in the central region compared with the peripheral cornea. Corneal epithelial cells differentiate gradually and lose their ability to proliferate as they migrate from the basal layer to the surface [17, 18]. Superficial cells undergo apoptosis and desquamate from the surface of the cornea [19]. From this point of view, superficial epithelial cells in the central and peripheral cornea of all age groups are at the same level (the last stage) of maturity. The present study confirms that there is no significant difference in superficial cell morphology, size or density between different regions or among different age groups.

With respect to basal epithelial cells, our study found a significant difference in cell density and area between the central and peripheral cornea in the youngest group (group A). This was in agreement with the age-related changes demonstrated in our study; wherein, peripheral basal epithelial cell density in group A was significantly higher than the other three groups, while central basal epithelial cell density revealed no significant age-related changes. Age-related changes of peripheral basal epithelial density were consistent with the age-related decrease in limbal epithelial cell density demonstrated in our previous study [20]. For the renewal and recovery of the cornea, epithelial cells migrate from the limbus, which contains corneal epithelial stem cells, to the peripheral, and finally to the central cornea [6]. The age-related decrease in limbal epithelial cell density might have an influence on the nearby peripheral transparent cornea.

Regional differences in cell density and size have clinical implications. The proliferative activity of epithelial cells has a gradient relationship with cell size [21, 22], since the smallest cell has the highest clone formation rate [23]. Basal epithelial cells in the limbus were significantly smaller than those in the central cornea, which coincided with the stronger proliferative ability of cells in the limbal epithelium [24, 25]. In group A, the smaller cell size and higher cell density in the peripheral cornea suggest that basal epithelial cells might have a stronger proliferative ability in childhood, with more potential for self-repair after injury. However, no difference was found between other groups, indicating that the central and peripheral corneal epithelium itself has the same proliferative capacity in adulthood. Therefore, the relatively quick repair of the epithelium in the peripheral cornea in adults, which was previously reported [6–8], is more likely to be attributed to the fact that the peripheral cornea is closer to the limbus; and the offspring cells of limbal stem cells may have taken less time to migrate there.

### Corneal stroma

In the current study, the background in the central cornea was uniformly dark, while the background in the peripheral cornea was hyper-reflective with more visible stromal fibers. Stromal collagen is invisible by CM in the normal central cornea, because collagen fibrils are compacted in regularly organized lamellae and light scattered by individual fibers is cancelled by the destructive interference from the scattered light of neighboring fibers. Thus, in the central cornea, keratocytes usually stood out against a clear dark background.

However, our study revealed that the background in the peripheral cornea appeared to be hyper-reflective with more visible stromal fibers. Previous studies have found that the collagen fibrils in the central cornea were orthogonally arranged between the neighboring lamellae, while they were organized in a circumferential annulus in the limbus [26–28]. Thus, collagen fibrils became irregularly arranged in the peripheral region of the cornea, which consequently increased light scattering [26–28]. In addition, previous studies found a sharp rise in both the size of collagen fibrils and number of lamellae in the peripheral cornea compared with the central region, which correlated with the thickening and stiffness of the peripheral cornea for the maintenance of corneal curvature [28–30]. For these reasons, we detected a relatively hyper-reflective background and more visible stromal fibers in the peripheral cornea; which indicate that both the optical and biomechanical properties in this region changed.

In age groups B and C, we found that the density of anterior stromal keratocytes was significantly higher in the central cornea compared with the peripheral region. This could be explained by the different inflexion points of the decline for the central and peripheral regions of the cornea (Fig. 4). In group A, keratocyte density of the anterior region was not significantly different compared with the central and peripheral regions. However, keratocyte density in the anterior peripheral region started to decline early from group A to B, while keratocyte density in the anterior central region started to decline later from group C to D. As a result, anterior keratocyte density in groups B and C was significantly higher in the central cornea compared with the peripheral region. Finally, anterior keratocyte density decreased in both regions of the cornea to the same level in group D. However, the changes we observe in keratocyte density with age were not significantly different (*P* > 0.05). This could be attributed to the limited sample size, considering that a significant age-related decrease in anterior and posterior keratocyte density was found in previous studies [11, 12].

Keratocytes play a crucial role in the repair of the corneal epithelium and stroma, and keratocytes in the anterior region near or at the site of damage underwent apoptosis quickly after epithelial or stromal injury. The surrounding healthy keratocytes initiated deoxyribonucleic acid (DNA) synthesis within two hours and migrated into the damaged area in three days, secreting collagenase to clean the debris of the injury and producing new collagen to rebuild the damaged area [31, 32]. In addition, keratocytes could secrete cytokines such as hepatocyte and keratinocyte growth factors to induce the proliferation of epithelial cells [33]. A higher density of keratocytes in the anterior central region found in the present study may facilitate the repair of damage, which might to some extent compensate for the weakness of the central cornea in less thickness, and subsequently reduce the possibility of perforation in the central cornea relatively.

### Corneal endothelium

The present study demonstrates that age-related degeneration in endothelial density and the percentage of hexagonal cells in both the central and peripheral cornea was consistent with previous studies [11, 13]. We found that the density of endothelial cells in the central cornea was not significantly different from that in the periphery. A major function of the corneal endothelium is to maintain corneal transparency by regulating corneal hydration. Previous studies have shown that for contact lens wearers, edema occurs more frequently in the central region of the cornea compared with the peripheral region [8]. In the current study, no significant differences in endothelial cell density between the central and peripheral region were found; thus, it is more reasonable to address this phenomenon from the perspective of oxygen supply. The major source of oxygen for the central and peripheral region is the tear and vascular network, respectively [1]. Wearing contact lenses interferes with oxygen regeneration in the tear film and restricts oxygen supply, which result in anaerobic respiration and induce lactic acid formation and accumulation in the central cornea; leading to increased intra-corneal osmolarity and central corneal edema [8].

We also found that the percentage of hexagonal cells in the endothelium was significantly lower in the peripheral cornea (46 ± 11%) compared with the central region of the cornea (50 ± 11%). It has been demonstrated that when endothelial cells were exposed to stress, especially along with cell loss, the remaining cells may lose their hexagonal shape and become irregular in shape and size. These changes can occur with age, after trauma and in long-term contact lens wearers [34]. Corneas with reduced hexagonal endothelial cells could not withstand trauma as well as corneas with normal levels of hexagonal endothelial cells [34]. However, it is uncertain whether a 4% decrease of hexagonal cells measured in the current study could significantly influence endothelial cell function or the optical properties of the cornea; which requires further studies.

One limitation of the current study is we chose the inferior peripheral cornea for examination, instead of observing all four quadrants. Therefore, the results of this study might not be extended to the other peripheral regions. The reason for choosing the inferior peripheral location was for the convenience and comfort of the subjects. Performing confocal microscopy on each quadrant would take considerably more time. The subjects feel uncomfortable with speculum on their eyes, and with stinging and tears they do not cooperate well with the examination. Therefore, we measured only one position of the periphery cornea. As it is easier for the subjects with speculum to look upwards and hold their eyes in that position, we chose the inferior peripheral location for examination.

Another limitation of the current study was that we were not able to analyze the corneal nerves and the dendritic cells because of the relatively lower resolution of the CM which used a halogen lamp as the light source. Its capability of recognizing corneal nerves and dendritic cells was significantly lower than CM that use a laser as a light source. Further studies could be performed with the laser scanning CM to assess the differences of corneal nerves and dendritic cells between the central and peripheral cornea.

## Conclusion

In conclusion, along with a generally consistent microstructure, some differences were found between the central and peripheral regions of the transparent cornea in healthy subjects. These differences provide a histological basis for further studies on the optical and biomechanical properties and pathological mechanisms of the central and peripheral regions of the cornea.

## Abbreviations

ANOVA, analysis of variance; BUT, tear film break up time; CM, confocal microscopy; DNA, deoxyribonucleic acid; LSD, significant difference; TMH, tear meniscus height.
